# Transcranial Doppler Based Cerebrovascular Reactivity Indices in Adult Traumatic Brain Injury: A Scoping Review of Associations With Patient Oriented Outcomes

**DOI:** 10.3389/fphar.2021.690921

**Published:** 2021-07-06

**Authors:** Alwyn Gomez, Logan Froese, Amanjyot Singh Sainbhi, Carleen Batson, Frederick A. Zeiler

**Affiliations:** ^1^ Section of Neurosurgery, Department of Surgery, Rady Faculty of Health Sciences, University of Manitoba, Winnipeg, MB, Canada; ^2^ Department of Human Anatomy and Cell Science, Rady Faculty of Health Sciences, University of Manitoba, Winnipeg, MB, Canada; ^3^ Biomedical Engineering, Faculty of Engineering, University of Manitoba, Winnipeg, MB, Canada; ^4^ Centre on Aging, University of Manitoba, Winnipeg, MB, Canada; ^5^ Division of Anaesthesia, Department of Medicine, Addenbrooke’s Hospital, University of Cambridge, Cambridge, United Kingdom

**Keywords:** cerebral autoregulation, cerebrovascular reactivity, scoping review, traumatic brain injury, transcranial Doppler, precision medicine

## Abstract

**Background:** Disruption in cerebrovascular reactivity following traumatic brain injury (TBI) is a known phenomenon that may hold prognostic value and clinical relevance. Ultimately, improved knowledge of this process and more robust means of continuous assessment may lead to advances in precision medicine following TBI. One such method is transcranial Doppler (TCD), which has been employed to evaluate cerebrovascular reactivity following injury utilizing a continuous time-series approach.

**Objective:** The present study undertakes a scoping review of the literature on the association of continuous time-domain TCD based indices of cerebrovascular reactivity, with global functional outcomes, cerebral physiologic correlates, and imaging evidence of lesion change.

**Design:** Multiple databases were searched from inception to November 2020 for articles relevant to the association of continuous time-domain TCD based indices of cerebrovascular reactivity with global functional outcomes, cerebral physiologic correlates, and imaging evidence of lesion change.

**Results:** Thirty-six relevant articles were identified. There was significant evidence supporting an association with continuous time-domain TCD based indices and functional outcomes following TBI. Indices based on mean flow velocity, as measured by TCD, were most numerous while more recent studies point to systolic flow velocity-based indices encoding more prognostic utility. Physiologic parameters such as intracranial pressure, cerebral perfusion pressure, Carbon Dioxide (CO2) reactivity as well as more established indices of cerebrovascular reactivity have all been associated with these TCD based indices. The literature has been concentrated in a few centres and is further limited by the lack of multivariate analysis.

**Conclusions:** This systematic scoping review of the literature identifies that there is a substantial body of evidence that cerebrovascular reactivity as measured by time-domain TCD based indices have prognostic utility following TBI. Indices based on mean flow velocities have the largest body of literature for their support. However, recent studies indicate that indices based on systolic flow velocities may contain the most prognostic utility and more closely follow more established measures of cerebrovascular reactivity. To a lesser extent, the literature supports some associations between these indices and cerebral physiologic parameters. These indices provide a more complete picture of the patient’s physiome following TBI and may ultimately lead to personalized and precise clinical care. Further validation in multi-institution studies is required before these indices can be widely adopted clinically.

## Introduction

The disruption of cerebral autoregulation (CA) following traumatic brain injury (TBI) has been known since the 1970s (Overgaard and Tweed, 1974; Cold and Jensen, 1978). At that time, evaluation of CA was cumbersome and involved perturbation of the patient’s blood pressure while low frequency measurement of cerebral blood flow (CBF) were obtained. This meant that assessment of dynamic changes in CA was limited and thus its utility in precision medicine limited.

Aaslid and colleagues first described transcranial doppler ultrasound (TCD) in 1982 as a non-invasive means of evaluating CBF through insonation of flow velocities (FV) in the basal arteries of the brain (Aaslid et al., 1982). Since then, the role of TCD in the management of TBI patients has grown substantially with widespread adoption in the neurocritical care setting. It has become one of the most commonly utilized methods for intracranial monitoring in the critically ill TBI patient, outside of ICP monitoring, and the most popular non-invasive cerebral monitoring modality for this population. While direct TCD measures, such as FV, have been measured for their association with secondary neurologic decline and global outcomes, derived TCD metrics have been developed to non-invasively estimate intracranial pressure (ICP), carbon dioxide (CO_2_) reactivity and even CA (Gomez et al., 2021). Unfortunately, early methods of evaluating CA using TCD, such as the Thigh Cuff Deflation Technique (TCDT) and Orthostatic Hypotension Test (OHT), were intermittent in nature since they still depended on induced changes in arterial blood pressure (ABP) (Aaslid, 1986; Aaslid et al., 1989; Steinmeier et al., 2002).

TCD was first described as a tool to continuously evaluate CA following TBI by Czosnyka and colleagues in 1996 (Czosnyka et al., 1996). In this study, they described a time-domain based mean flow index (Mx), a continuously updating Pearson correlation coefficient between the natural fluctuations in cerebral perfusion pressure (CPP), equal to the difference between ABP and ICP, and mean FV through the middle cerebral artery (MCA) as measured by TCD. This method used mean FV as a surrogate measure for CBF with CPP used as the driving force in order to continuously interrogate cerebrovascular reactivity. It should be noted that cerebrovascular reactivity and CA are not synonymous as vascular reactivity can occur outside the limits of autoregulation (Varsos et al., 2014). Being a correlation coefficient, Mx ranged from +1 to −1 with a more negative correlation being associated with intact cerebrovascular reactivity and a more positive coefficient being associated with disrupted reactivity. In their study of 82 moderate and severe TBI patients, they found a correlation between the state of cerebrovascular reactivity, as measured by Mx, and 6-month outcomes (Czosnyka et al., 1996). Of note, for metrics to be designated as a CA measure, it must have some pre-clinical validation it its ability to measure aspects of the Lassen autoregulatory curve. To date, TCD-based metrics have not received such validation. As such, such measures are referred to as cerebrovascular reactivity metrics, as opposed to CA measures, as the provide surrogate assessments of cerebral vessel vasomotion, but have yet to be validated as CA measures. Subsequently, through the remainder of this article, such TCD-based measures will be referred to as cerebrovascular reactivity metrics.

Since then, a significant amount of research has been undertaken to examine the association between continuous TCD based indices of cerebrovascular reactivity and outcomes following TBI (It should be noted that cerebrovascular reactivity and CA are not entirely interchangeable as cerebrovascular reactivity can occur outside the limits of autoregulation. Cerebrovascular reactivity is the broader term that describes the physiologic process that is measured by these indices). Slightly modified indices that used the diastolic and systolic FV were examined (Dx and Sx, respectively) along with indices that utilized ABP, as opposed to CPP, as the driving force (Mx_a, Dx_a, and Sx_a). Table 1 summarizes these indices and their derivation. Notably, those that utilize ABP instead of CPP open the door to the entirely non-invasive measurement of cerebrovascular reactivity (Zeiler et al., 2018b; Zeiler et al.,2019b; Zeiler and Smielewski, 2018; Gomez et al., 2020). This has the potential to expand their application to the neurocritical care of patient populations that do not typically have ICP monitoring as well as to the outpatient setting. While not yet adopted widely in clinical practice, these indices have an ever-growing body of evidence supporting their association with outcomes following TBI. The development of these indices has renewed the intertest in leveraging measures of cerebrovascular reactivity in the development of personalized treatments following TBI. Further to this, adoption of this continuous non-invasive cerebrovascular reactivity assessment has expanded outside of TBI, including recent work in subarachnoid haemorrhage and general operative populations (Budohoski et al., 2012b; Klein et al., 2019). Thus, to aid with the development of further prospective studies in both TBI and non-TBI cohorts, a comprehensive understanding of the association between TCD based continuous time-domain cerebrovascular reactivity indices with patient-oriented outcomes is warranted. Given that the majority of the literature to date is focused in the TBI populations, the natural first step is to provide a comprehensive scoping overview of the association between TCD based cerebrovascular reactivity indices with: A. global patient outcomes, B. other cerebral physiologic correlates, and C. lesion change/progression on serial imaging. Thus, the aim of this study was to perform a systematically conducted review of the literature to evaluate the association between these continuous time-domain TCD based indices of cerebrovascular reactivity and the above outcomes, in adult moderate/severe TBI. In doing so, a better understanding of the role these indices play in describing the post-TBI physiome may be developed.

**TABLE 1:** Various transcranial Doppler based Indices of cerebrovascular reactivity with their component signals and derivation.

Index	Surrogate for cerebral blood flow	Surrogate for driving force	Signal averaging (s)	Calculation windows (s)	Update frequency (min)
Mx	Mean CBFV	CPP	10	300	1
Sx	Systolic CBFV	CPP	10	300	1
Dx	Diastolic CBFV	CPP	10	300	1
Mx_a	Mean CBFV	ABP	10	300	1
Sx_a	Systolic CBFV	ABP	10	300	1
Dx_a	Diastolic CBFV	ABP	10	300	1

ABP, Arterial blood pressure; CBFV, Cerebral blood flow velocity (measured by transcranial Doppler); CPP, Cerebral perfusion pressure.

## Methods

A systematically conducted scoping review of the available literature was conducted based on the methodological framework described by Arksey and O’Malley (2005). The data was reported in line with the Preferred Reporting Items for Systematic Reviews and Meta-Analyses Extension for Scoping Reviews (PRISMA-ScR; Tricco et al., 2018). The search strategy and methodology used here is similar to other systematically conducted scoping reviews published by our group (Froese et al., 2020a, Froese et al.,2020b, Froese et al.,2020c; Hasen et al., 2020).

The review questions and search strategy were decided upon by the senior author (FAZ) and primary author (AG)

### Search Questions, Populations, and Inclusion/Exclusion Criteria

The question of this systematically conducted scoping review was: What is the association of continuous time-domain TCD based indices of cerebrovascular reactivity, with: A. global functional outcomes, B. cerebral physiologic correlates, and C. imaging evidence of lesion change following moderate-to-severe TBI?

All English language studies, either prospective or retrospective, with 20 or more adult (age 18 years or older) moderate and severe TBI patients, were included. Moderate TBI is defined as an admission Glasgow coma scale (GCS) of 9–12 while a GCS of 3–8 defines severe TBI.

Only continuous time-domain TCD based cerebrovascular reactivity metrics were of interest, excluding both intermittent techniques (Zeiler et al., 2017a) and those based in frequency-domain (i.e., transfer-function techniques; Czosnyka et al., 2008). The eligible time-domain TCD based cerebrovascular reactivity indices of interest were Mx, Sx, Dx, Mx_a, Sx_a, and Dx_a. Table 1 outlines the components of these indices and their derivation.

The primary outcome of interest was the statistically significant association between these indices and morbidity/mortality following TBI. Secondary outcomes included statistically significant associations with severity of injury and age. Additionally, associations with various cerebral physiologic parameters such as ICP, CPP, brain tissue oxygenation (PbtO_2_), and CO_2_ reactivity were also examined. Give that the pressure reactivity index (PRx) has become a widely accepted continuous measures of cerebrovascular reactivity (Czosnyka et al., 1997; Zeiler et al., 2018d), its associations with TCD based indices were also collected. Associations between TCD based indices and more novel ICP based indices of cerebrovascular reactivity, such as PAx and RAC, were excluded as these indices are not as well established despite their evidence of their prognostic utility in TBI (Zeiler et al., 2017b). Table 2 outlines the cerebral physiologic parameters that were examined for their association with TCD based indices. All parameters selected as secondary outcomes are known to be associated with global outcomes and/or are physiologic targets in guideline based management following moderate to severe TBI (Carney et al., 2017; Zeiler et al., 2018d; Hawryluk et al., 2019).

**TABLE 2:** Outline of Cerebral Physiologic Parameters of Interest

Cerebral Physiologic Parameter	Description	Clinical relevance
Intracranial pressure (ICP)	An invasively measured physiologic parameter obtained through either the placement of an intraparenchymal probe or placement of an intraventricular catheter. It represents the pressure experienced by the brain	Following TBI values greater than 20–22 mmHg are associated with worse outcomes. Current guideline-based management recommends maintain ICP less than 22 mmHg post injury (Carney et al., 2017)
Cerebral perfusion pressure (CPP)	This is a derived physiologic parameter equal to the difference between ABP and ICP. It represents the net pressure gradient that drives oxygen delivery to the brain	Following TBI values greater than 70 mmHg and less than 60 mmHg are associated with worse outcomes. Current guideline-based management recommends maintain CPP between 60 and 70 mmHg post injury (Carney et al., 2017)
Brain tissue oxygenation (PbtO2)	An invasively measured physiologic parameter obtained through the placement of an intraparenchymal Clark electrode. It measures extracellular oxygen content and is thought to reflect cerebral oxygenation.	Following TBI values less than 20 mmHg are associated with worse outcomes. Current guideline-based management recommends maintain a PbtO2 greater than 20 mmHg (Hawryluk et al., 2019)
CO2 reactivity	This is the propensity of the brains vasculature to dilate in the setting of elevated PaCO2 and constrict in the setting of a reduced PaCO2. It can be measured in numerous methods, including TCD, and can be leveraged in the acute management of TBI	Following TBI, if CO2 reactivity is intact, elevated ICP can be treated transiently with hyperventilation to reduce PaCO2 which reduces CBV and results in a decrease in ICP (Carney et al., 2017)
Pressure reactivity index (PRx)	This derived invasive index is a moving Pearson correlation coefficient between ICP and ABP. It ranges in value from −1.0 to +1.0 and is thought to represent cerebrovascular reactivity with higher correlation (and therefore higher vales) being associated with disrupted cerebrovascular reactivity	Recently it has been found that following moderate-to-severe TBI PRx values of +0.35 are associated with increased morbidity and mortality (Zeiler et al., 2018d)

ABP, arterial blood pressure; CBV, cerebral blood volume; CO2, carbon dioxide; CPP, cerebral perfusion pressure; ICP, intracranial pressure; mmHg, millimetres of mercury; PaCO2, partial pressure of carbon dioxide; PbtO2, brain tissue oxygenation; PRx, pressure reactivity index; TBI, traumatic brain injury; TCD, transcranial doppler.

Studies relating to TCD time-domain based cerebrovascular reactivity measures and imaging changes were searched for. Specifically, studies examining the association of these indices with changes in CT score (Marshall, Rotterdam, Helsinki, or Stockholm), midline shift, and hematoma volume as well as the development on new lesions were all considered relevant.

Exclusion criteria for studies were the following: non-English, non-human, non-TBI, mild TBI, paediatric cohorts, non-time-domain TCD based indices, non-continuous TCD metrics, cohort < 20 TBI patients, or no relevant outcome (functional or physiologic) association. Review studies and meta-analysis were also excluded from consideration.

### Search Strategy

BIOSIS, Cochrane Library, EMBASE, MEDLINE, and SCOPUS were searched from inception to November 2020 using individualized search strategies for each database. The search strategy for SCOPUS can be seen in Supplementary Appendix A with similar strategies used for each of the other databases. Finally, the reference lists of each article were reviewed to ensure no studies were missed. Search results were then combined, and deduplication was performed.

### Study Selections

Using two reviewers (AG and LF) a two-step review of all articles returned by our search strategies was performed. In the first filter phase, each reviewer independently screened all studies identified using the above-described search strategy and determined if they met the inclusion criteria based on their title and abstract. The resulting list of studies was then passed through the second filter phase where once again each reviewer independently determined if the studies met the inclusion criteria, but this time based on the full text. Any discrepancies between the two reviewers were resolved by a third party (FAZ).

### Data Collection

Data was extracted from the selected articles and compiled into various data fields. These fields included the following: number of patients, study design, institution, mean age, mean GCS on presentation, number of male patients, additional patient characteristics, goals of the study, indices examined, duration of insonation, outcomes evaluated, key results, and conclusions.

### Bias Assessment

Given the goal of this review was to provide a comprehensive scoping overview of the available literature, a formal bias assessment was not conducted.

### Statistical Analysis

Due to the heterogeneity of the results/study design no meta-analysis was performed.

## Results

### Search Results and Study Characteristics

**FIGURE 1:**
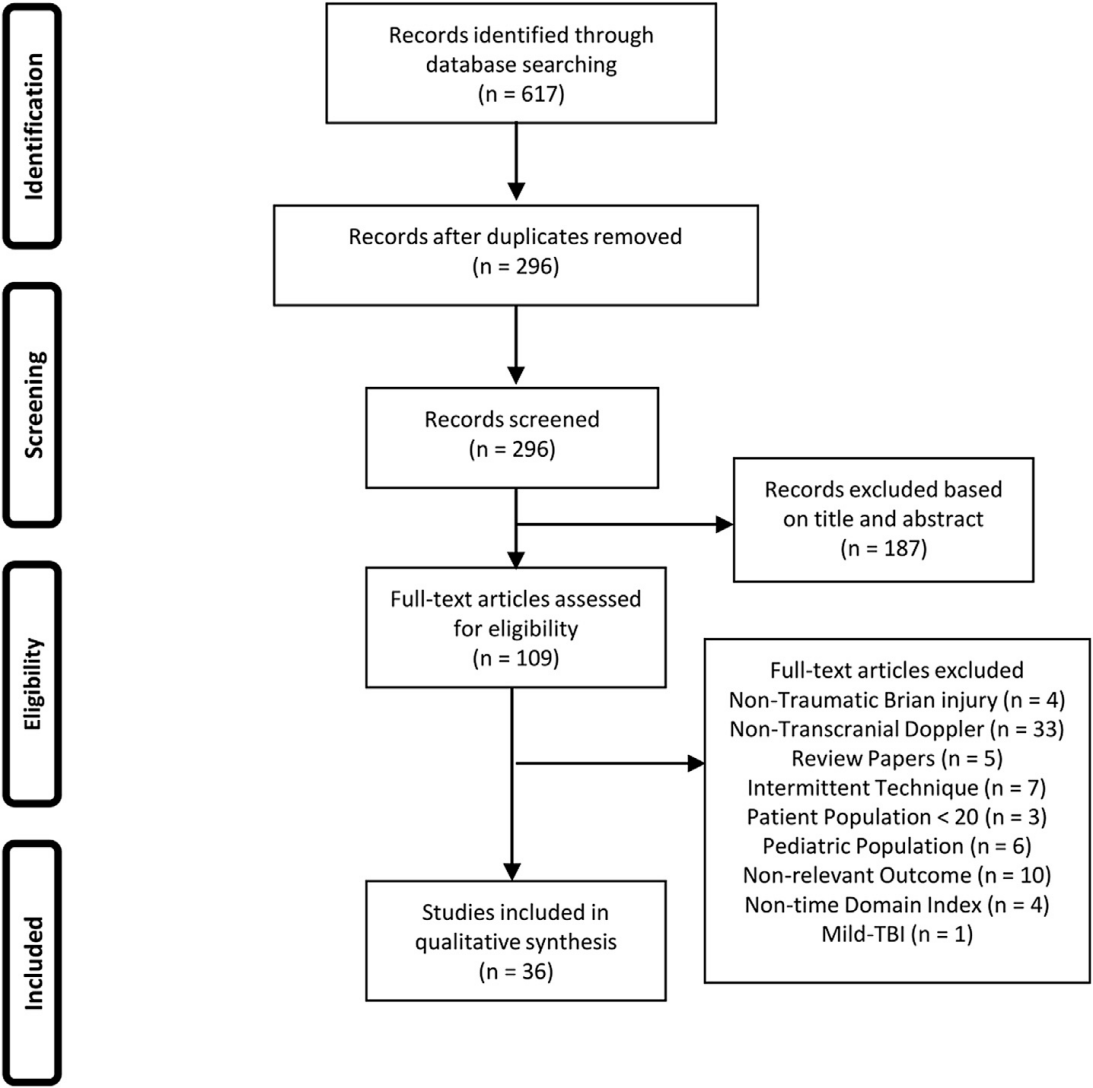
A PRISMA flow-diagram of the systematically conducted scoping review of the literature.

The results of the search and filtration strategy can be seen in Figure 1. Overall, the search strategy identified 617 articles with 296 remaining following deduplication. Following the first filtration stage, based on article title and abstract, 187 articles were removed for not fitting into the inclusion/exclusion criteria. Full text documents were reviewed on the remaining 109 articles in the second filtration phase with 73 articles being found to not meet the inclusion/exclusion criteria for this study. This left 36 articles to be included in this scoping review. Figure 1 provides the PRISMA flow-diagram.

Table 3 summarizes general characteristics of each study while Table 4 outlines the key results, conclusions, and limitations of each study. Mx was the most common time-domain TCD index that was studied, with all but one study reporting on it (Czosnyka et al., 2005). It was also the only index with evidence showing that it worsened with injury severity (Czosnyka et al., 1996; Czosnyka et al., 1999; Czosnyka et al., 2000) and with advanced age (Czosnyka et al., 2005; Czosnyka et al., 2008). The next most common index was Mx_a with it being measure in nine studies (Lang et al., 2003a; Lang et al., 2003b; Lewis et al., 2007; Sorrentino et al., 2011; Budohoski et al., 2012c; Liu et al., 2015; Zeiler et al., 2017b; Zeiler et al., 2018a; Zeiler et al., 2018c). Notably, Mx, and Mx_a have been found to be strongly associated with one another (Schmidt et al., 2003a; Lewis et al., 2007; Sorrentino et al., 2011). The remainder of time-domain TCD based indices were examined only in a minority of studies. The number of patients with TCD recordings included in each study varied from as few as 20 TBI patients to as many as 347 (Schmidt et al., 2016b; Zeiler et al., 2018e). It should be noted that only five of the 36 articles did not report on data from patients at the Addenbrooke’s Hospital in Cambridge, United Kingdom with the largest of these studies only including 40 TBI patients (Lang, 2003; Lang et al., 2003a; Lang et al., 2003b; Schmidt et al., 2016a; Schmidt et al., 2016b).

**TABLE 3:** Study Characteristics of Included Studies.

Reference	Number of Patients	Institution	Study design	Mean age (Range)	Mean admission GCS (Range)	Number of male Patients	Additional patient characteristics	Duration of insonation	Relevant indices evaluated
Global functional outcomes									
Budohoski et al. (2012a)	201	Addenbrooke’s Hospital, United Kingdom	Retrospective analysis of prospectively collected data	23 (11–78)	6	157	None reported	Not reported	Mx/PRx
Budohoski et al. (2012c)	300	Addenbrooke’s Hospital, United Kingdom	Retrospective analysis of prospectively collected data	29	6	226	None reported	Not reported	Mx/Sx/Dx/ Mx_a/Sx_a/ Dx_a
Czosnyka et al. (1996)	82	Addenbrooke’s Hospital, United Kingdom	Retrospective analysis of prospectively collected data	36 (7–75)	6 (3–13)	55	No patients with craniectomy	Daily for 20 min -2 h	Mx/Sx
Czosnyka et al. (1997)	82	Addenbrooke’s Hospital, United Kingdom	Retrospective analysis of prospectively collected data	37 (6–75)	7 (3–13)	55	No patients with craniectomy	Not reported	Mx/PRx
Czosnyka et al. (1998)	82	Addenbrooke’s Hospital, United Kingdom	Retrospective analysis of prospectively collected data	36 (7–75)	6 (3–13)	55	None reported	Daily for 20 min–2 h	Mx/PRx
Czosnyka et al. (1999)	98	Addenbrooke’s Hospital, United Kingdom	Retrospective analysis of prospectively collected data	38 (14–76)	< 8 (3–13)	68	None reported	Daily for 20 min–4 h	Mx
Czosnyka et al. (2000)	166	Addenbrooke’s Hospital, United Kingdom	Retrospective analysis of prospectively collected data	None reported	None reported	Not reported	None reported	Daily 30 min–2 h	Mx
Czosnyka et al. (2001)	187	Addenbrooke;s Hospital, United Kingdom	Retrospective analysis of prospectively collected data	36 (6–75)	6 (3–13)	143	31% SDH 28% ICH 10% EDH 11% DAI 57% Brain Swelling 38% MLS 25% tSAH	Daily for 20 min–2 h	Mx
Czosnyka et al. (2002)	188	Addenbrooke’s Hospital, United Kingdom	Retrospective analysis of prospectively collected data	None reported	None reported	Not reported	None reported	Daily for 30 min–2 h	Mx/PRx
Czosnyka et al. (2005)	358	Addenbrooke’s Hospital, United Kingdom	Retrospective analysis of prospectively collected data	None reported	None reported	Not reported	237 patients with TCD data	Daily for 20 min–2 h	Sx/PRx
Czosnyka et al. (2008)	50	Addenbrooke’s Hospital, United Kingdom	Retrospective analysis of prospectively collected data	31 (17–75)	6 (3–13)	34	15% SDH 20% ICH 10% EDH 10% DAI 50% Brian Swelling 22% MLS 25% tSAH	Daily for 20 min–2 h	Mx
Lang et al. (2003a)	37	Christian-Albrechts-Universität, Ger. and University of Sydney, Aus.	Retrospective analysis of prospectively collected data	41	8.4	30	6 EDH 23 SDH 19 Contusion 2 DAI	Not reported	Mx/Mx_a
Lang et al. (2003b)	25	Christian-Albrechts-Universität, Ger. and University of Sydney, Aus.	Retrospective analysis of prospectively collected data	38 (16–58)	7.1	18	None reported.	18 min	Mx_a
Lewis et al. (2007)	151	Addenbrooke’s Hospital, United Kingdom	Retrospective analysis of prospectively collected data	36 (16–75)	6 (3–13)	121	30% SDH 25% ICH 13% DAI 59% Brain Swelling 32% MLS 23% tSAH	Daily for 20 min–2 h	Mx/Mx_a
Lewis et al. (2012)	187	Addenbrookes Hospital,United Kingdom	Retrospective analysis of prospectively collected data	None reported	None reported	Not reported	None reported	Not reported	Mx
Liu et al. (2015)	288	Addenbrooke’s Hospital, United Kingdom	Retrospective analysis of prospectively collected data	33	6	Not reported	None reported	Daily for 20 min–1 h	Mx/Mx_a
Radolovich et al. (2011)	293	Addenbrooke’s Hospital, United Kingdom	Retrospective analysis of prospectively collected data	37 (13–78)	6	Not reported	None reported	Daily for 10 min–3 h (mean 32 min)	Mx
Schmidt et al. (2016a)	30	Chemnitz Medical Centre, Ger.	Retrospective analysis of prospectively collected data	51.4	None reported	Not reported	23 patients with TBI 7 patients with other cerebral disease	Not reported	Mx/PRx
Schmidt et al. (2016b)	41	Chemnitz Medical Centre, Ger	Retrospective analysis of prospectively collected data	52 (18–77)	None reported	28	20 TBI patients 21 patients with other cerebral disease	Not reported	Mx/PRx
Sorrentino et al. (2011)	248	Addenbrooke’s Hospital, United Kingdom	Retrospective analysis of prospectively collected data	28 (3–78)	6 (3–15)	195	None reported	Daily for 20 min–2 h	Mx/Mx_a
Zeiler et al. (2017b)	37	Addenbrooke’s Hospital, United Kingdom	Retrospective analysis of prospectively collected data	33 (16–76)	8 (3–14)	Not reported	None reported	Two separate recordings of 60 min	Mx/Sx/Dx/ Mx_a/Sx_a/Dx_a/PRx
Zeiler et al. (2018a)	281	Addenbrooke’s Hospital, United Kingdom	Retrospective analysis of prospectively collected data	33.5	6	231	None reported.	30 min or greater	Mx/Sx/Dx/ Mx_a/Sx_a/Dx_a/PRx
Cerebral physiologic correlates									
Czosnyka et al. (2003)	345 (243 TBI)	Addenbrooke’s Hospital, United Kingdom	Retrospective analysis of prospectively collected data	None reported	None reported	Not reported	The cohort included: 14 Healthy Volunteers 243 TBI Patients 15 aSAH Patients 15 aSAH Patients 38 Patients with Carotid Stenosis 35 Patients with Hydrocephalus	Daily for 20 min–2 h	Mx
Haubrich et al. (2011)	30	Addenbrooke’s Hospital, United Kingdom	Prospective Observational	39	None reported	Not reported	None reported.	20 min at normocapnia 30 min at hypocapnia	Mx
Haubrich et al. (2012)	29	Addenbrooke’s Hospital, United Kingdom	Prospective Observational	39	None reported	Not reported	None reported	20 min at normocapnia 30 min at hypocapnia	Mx
Lang (2003)	40	Christian-Albrechts-Universität, Ger. and University of Sydney, Aus.	Retrospective analysis of prospectively collected data	40 (16-78)	8 (3-15)	32	6 EDH 23 SDH 21 contusion 4 DAI 2 IVH	Not reported	Mx
Lewis et al. (2014)	21	Addenbrooke’s Hospital, United Kingdom	Retrospective analysis of prospectively collected data	24 (17-71)	4 (3-11)	17	10 Focal Haemorrhage 12 DAI 7 Brain Swelling 29 Plateau Waves	Not reported	Mx
Liu et al. (2016)	24	Addenbrooke’s Hospital, UK	Retrospective analysis of prospectively collected data	None reported	None reported	Not reported	30 Plateau Waves	Not reported	Mx /Mx_a
Schmidt et al. (2003a)	145	Addenbrooke’s Hospital, United Kingdom; Munich-Bogenhauses Medical Centre, Ger.; Munich-Schwabing Medical Centre, Ger.; Chemnitz Medical Centre, Ger.; and Frankfurt Medical Centre, Ger.	Retrospective analysis of prospectively collected data	35 (3-76)	None reported	111	The cohort included 135 TBI patients (39 SDH, 35 ICH, 68 Brain Oedema) and 10 haemorrhagic stroke patients	Not reported	Mx/Mx_a
Schmidt et al. (2003b)	96	Addenbrooke’s Hospital, United Kingdom	Retrospective analysis of prospectively collected data	31 (16-76)	6 (3-14)	84	27 patients with MLS	Daily for 20 min-2 h	Mx
Schmidt et al. (2009)	53	Addenbrooke’s Hospital, United Kingdom and Chemnitz Medical Centre, Ger.	Retrospective analysis of prospectively collected data	None reported	None reported	Not reported	None reported	Not reported	Mx/Mx_a
Schmidt et al. (2012)	62	Addenbrooke’s Hospital, United Kingdom and Chemnitz Medical Centre, Ger.	Retrospective analysis of prospectively collected data	None reported	None reported	Not reported	None reported	Daily for 20 min–2 h	Mx
Zeiler et al. (2018c)	40	Addenbrooke’s Hospital, United Kingdom	Retrospective analysis of prospectively collected data	31.1	5	Not reported	None reported	30 min to 1 h	Mx/Sx/Dx/ Mx_a/Sx_a/Dx_a/PRx
Zeiler et al. (2018e)	347	Addenbrooke’s Hospital, United Kingdom	Retrospective analysis of prospectively collected data	33.7	250	250	None reported.	30 min–3.26 h	Mx_a/Sx_a/ PRx
Zhang et al. (2016)	31	Addenbrooke’s Hospital, United Kingdom	Prospective Observational	None reported	None reported	Not reported	None reported	Not reported	Mx
Zweifel et al. (2008)	398	Addenbrooke’s Hospital, United Kingdom	Retrospective analysis of prospectively collected data	33 (16–79)	7 (3–13)	314	17 Craniectomy 298 with TCD data	Not reported	Mx/PRx

ABP, arterial blood pressure; aSAH, aneurysmal subarachnoid haemorrhage; CPP, cerebral perfusion pressure; DAI, diffuse axonal injury; Dx, diastolic flow index with CPP; Dx_a, diastolic flow index with ABP; EDH, epidural hematoma; ICH, intracerebral haemorrhage; IVH, intraventricular haemorrhage; MLS, midline shift; Mx, mean flow index with CPP; Mx_a, mean flow index with ABP; PRx, pressure reactivity index; SDH, subdural hematoma; TCD, transcranial doppler; tSAH, traumatic subarachnoid haemorrhage; Sx, systolic flow index with CPP; Sx_a, systolic flow index with ABP.

**TABLE 4:** Study goals, findings and limitations of included studies.

Reference	Relevant Goals of the Study	Key Relevant Results	Conclusion	Study Limitation
Global Functional Outcomes				
Budohoski et al. (2012a)	Primary: To assess the association of Mx with functional outcomes. Secondary: To assess the association of Mx with PRx.	Mx was significantly different between those that had unfavourable outcomes and those that had favourable outcomes (0.09 ± 0.26 vs. −0.03 ± 0.25, *p* = 0.002). Mx was significantly different in those that died and those that survived (0.12 ± 0.29 vs. 0.01 ±0.25, *p* = 0.018). Mx and PRx correlated well (r = 0.58, *p* < 0.01)	Mx was significantly lower in those with good functional outcomes and those that survived. Mx and PRx covary with one another	ABP, ICP and GCS were all significantly different between those that survived and those that died, and GCS and ABP were significantly different between those with favourable and unfavourable functional outcomes, but this was not controlled for when assessing the association with outcomes and Mx. Single institution dataset
Budohoski et al.( 2012c)	Primary: To assess the prognostic utility of Mx, Sx, Dx, Mx_a, Sx_a, and Dx_a.	Mx (F = 16.56, *p* = 0.00006), Sx (F = 20.11, *p* = 0.00001), Dx (F = 7.07, *p* = 0.008), Mx_a (F = 8.88, *p* = 0.003), and Sx_a (F = 12.49, *p* = 0.0005) were all able to discriminate between patients with favourable and unfavourable functional outcomes at follow up. Mx (F = 6.93, *p* = 0.009), Sx (F = 13.10, *p* = 0.0003) and Sx_a (F = 5.32, *p* = 0.02) were all able to discriminate between those that survived and those that did not at follow up	Mx, Sx, Dx, Mx_a, and Sx_a were able to discriminate between those with favourable and unfavourable functional outcomes with Sx having the strongest predictive value Mx, Sx, and Sx_a were able to discriminate between survival and death with Sx having the strongest predictive value	ICP and admission GCS were also found to be able to discriminate between favourable vs. unfavourable and death vs. survival but no multivariant analysis was performed to identify if the TCD indices had prognostic value independent of ICP and admission GCS Single institution dataset
Czosnyka et al. (1996)	Primary: To assess the association of Mx/Sx with global outcomes. Secondary: To assess the association of Mx/Sx with severity of injury, age, CPP and ICP	Mx (r = 0.41, *p* < 0.0002) and Sx (r = 0.48, *p* < 0.00009) correlated with 6-month GOS Mx (r = −0.34, *p* < 0.0025) and Sx (r = −0.38, *p* < 0.0008) correlated with admission GCS	Mx and Sx correlate with both injury severity and functional outcome	No multivariate analysis performed Single institution dataset
Czosnyka et al. (1997)	Primary: To assess the prognostic ability of PRx. Secondary: To assess the covariance of PRx and Mx	Mx positively correlated with PRx (r = 0.63, *p* < 0.000001) Mx (r = 0.41, *p* < 0.0002) correlated with 6-month GOS. Median Mx significantly differed in those with a favourable vs. unfavourable outcome (−0.26 vs. 0.03, *p* < 0.00008)	There is good correlation between Mx and PRx in non-craniectomy patients Mx was associated with functional outcomes at 6 months	No multivariate analysis performed. Single institution dataset
Czosnyka et al. (1998)	Primary: To assess the prognostic ability of PRx. Secondary: To assess the correlation of Mx and PRx	Mx (r = 0.41, *p* < 0.0002) correlated with 6-month GOS Mx positively correlated with PRx (r = 0.63, *p* < 0.000001)	There is good correlation between Mx and PRx in non-craniectomy patients Mx was associated with functional outcomes at 6 months	No multivariate analysis performed Single institution dataset previously reported on
Czosnyka et al. (1999)	Primary: To assess the correlation Mx with functional outcomes. Secondary: To assess the association of Mx with admission GCS, CPP and ICP	Mx correlated with 6-month GOS (r = 0.39, *p* < 0.05) Mx correlated with admission GCS (r = −0.28, *p* < 0.05) Mx correlated with ICP and CPP (r = 0.45 and r = −0.34, *p* < 0.05)	Mx was associated with functional outcomes at 6 months and injury severity Mx was associated with both ICP and CPP	No multivariate analysis performed so unclear if Mx association with outcome is mediated through association with injury severity, ICP, or CPP Single institution dataset
Czosnyka et al. (2000)	Primary: To assess the association of Mx with global functional outcomes Secondary: To assess the association of Mx with CPP	Mx was significantly greater in those with an unfavourable outcome (*p* < 0.001) Mx correlated with 6-month GOS and admission GCS (ANOVA, F value 17 and 15, *p* < 0.05) Mx depended on CPP (ANOVA, *p* < 0.0001) and became positive at CPP < 60 mm Hg	Mx was associated with functional outcomes at 6 months and injury severity Mx was associated with CPP with a lower threshold of CPP at 60 mm Hg	No multivariate analysis performed so unclear if Mx association with outcome is mediated through association with injury severity or CPP Single institution dataset
Czosnyka et al. (2001)	Primary: To assess the association of Mx with ICP and CPP Secondary: To assess the association of Mx with 6-month outcomes	The relationship between Mx and CPP is characterized by a U-shaped curve Mx worsened with increasing ICP with the steepest rise between 20 and 30 mm Hg Those with a favourable outcome at 6 months had a lower mean Mx (−0.06 ± 0.26 vs. 0.15 ± 0.31, *p* < 0.00002), lower mean ICP (17.0 ± 8.9 vs. 23.0 ± 13, *p* < 0.003), higher GCS on admission (6 vs. 4, *p* < 0.0018) and younger age (27 vs. 32, *p* < 0.015)	Mx has a U-shaped relationship with CPP and worsens with increasing ICP A favourable outcome was associated with lower Mx, lower ICP, Higher admission GCS, and younger age	No multivariate analysis performed so unclear if association of Mx with outcomes is mediated through ICP, CPP, admission GCS, age etc. Single institution dataset
Czosnyka et al. (2002)	Primary: To assess the association of Mx and PRx with outcomes Secondary: To identify a threshold value for Mx	Those with a favourable outcome had a lower mean Mx (−0.06 ± 0.26 vs. 0.15 ± 0.31, *p* < 0.00002), lower mean ICP (17.0 ± 8.9 vs. 23.0 ± 13, p < 0.003), higher GCS on admission (6 vs. 4, *p* < 0.0018) Mx (r = −0.2592, p < 0.0001), PRx (r = 0.278, *p* < 0.0001), ICP (r = −0.195, *p* < 0.01) and GCS (r = −0.18, *p* < 0.014) were all correlated with 6-month GOS Thresholds of Mx (0.23) and PRx (0.31) were found by identifying where the Kruskal-Wallis test reach its max) Mortality in those above thresholds consistently was 47% while those below threshold had a mortality of 11% (*p* < 0.0001) PRx and Mx correlated significantly (r = 0.57, *p* < 0.00001)	Mx is associated with 6-month outcomes with an increased mortality at Mx > 0.23	No multivariate analysis performed so unclear if association of Mx with outcomes is mediated through ICP, CPP, admission GCS, age etc. Single institution dataset No assessment of threshold outside of dataset from which it was developed
Czosnyka et al. (2005)	Primary: To assess the association of Sx with functional outcome Secondary: To assess the association of Sx with age	Sx not associated with 6-month GOS in multiple regression models Sx was significantly worse with age (r = 0.26, *p* = 0.002)	Sx was not found to be associated with outcome through multiple regression but cerebrovascular reactivity did worsen with age	Single institution dataset Sx was not evaluated in the entire study population due to incomplete TCD data leaving room for a selection bias
Czosnyka et al. (2008)	Primary: To assess the association of Mx with functional outcomes Secondary: To assess the association of Mx with age, ICP, CPP and admission GCS	Those with a favourable outcome had a lower Mx than those with an unfavourable outcome (−0.12 ± 0.28 vs. 0.21 ± 0.35, *p* = 0.0062) independent of age Mx was worse with age (r = 0.30, *p* = 0.034) Mx correlated positively with mean ICP (r = 0.31, *p* = 0.028) and negatively with CPP (r = −0.33, *p* = 0.016) There was no association with Mx and GCS on admission	Mx was found to correlate with functional outcomes independent of age Cerebrovascular reactivity, as measured by Mx, worsened with age and ICP while improving with increasing CPP. It was not associated with injury severity	Multivariate analysis did not account for CPP and ICP Single institution dataset
Lang et al. (2003a)	Primary: To assess the association of Mx and Mx_a with functional outcomes at discharge	Mx_a was associated with outcome ( r = −0.42, *p* < 0.05) Mx was associated with outcome (r= −0.56, *p* < 0.01) Mx and Mx_a did not correlated with ABP, CPP, ICP and CBFV	Mx and Mx_a are associated with functional outcomes at discharge	No long-term follow-up Single institution dataset No indication of duration of insonation
Lang et al. (2003b)	Primary: The incidence of hemispheric asymmetry (a difference of Mx_a > 0.2) and its association with outcome Hemispheric asymmetry had no impact on outcomes at discharge	12 of the 25 patients had a hemispheric asymmetry in Mx_a	Hemispheric asymmetry in cerebrovascular reactivity is relatively common following TBI but its impact on outcomes is unclear	No long-term follow-up Single institution dataset Very short duration of insonation
Lewis et al. (2007)	Primary: To assess the association of Mx and Mx_a with functional outcomes Secondary: To assess the covariance of Mx and Mx_a	There was no statistical association between outcome and Mx_a Mx was significantly different in those with a favourable vs. unfavourable outcome (−0.072 ± 0.21 vs. 0.12 ± 0.24, *p* = 0.007) Mx and Mx_a were associated with each other (r = 0.71, *p* < 0.05)	While Mx was associated with functional outcomes Mx_a was not Mx and Mx_a were found to be associated with one another	No multivariate analysis performed so unclear if association of Mx with outcomes is mediated through ICP, CPP, admission GCS, age etc. Single institution dataset
Lewis et al. (2012)	Primary: To assess the association of Mx with functional outcome Secondary: To assess the relationship between Mx and CPP	Mx was statistically different across GOS at 6 months (F = 5.39, *p* = 0.0013) Mx was able to discriminate between those with a favourable and unfavourable functional outcome (F = 14.2, *p* = 0.0002) Plotting Mx vs. CPP shows a U-shaped curve indicating a CPP at which Mx is at a minimum	Mx is associated with functional outcomes There is a U-shaped relationship between Mx and CPP indicating the possibility to identify a CPPopt	No multivariate analysis performed so unclear if association of Mx with outcomes is mediated through ICP, CPP, admission GCS, age etc. No reporting on the relationship between CPP and Mx in individual patients
Liu et al. (2015)	Primary: To assess the association of Mx Mx_a with functional outcomes	Mx was significantly different in those with favourable vs. unfavourable outcomes (−0.04 ± 0.29 vs. 0.09 ± 0.28 respectively, *p* < 0.0001, F value=15.38, AUC = 0.647) Mx_a was significantly different in those with favourable vs. unfavourable outcomes (0.18 ± 0.24 vs. 0.26 ± 0.21, respectively, p=0.002, F value = 10.08, AUC = 0.627)	Mx_a and Mx were both able to discriminate favourable vs. unfavourable functional outcomes but Mx had the stronger association with outcomes than Mx_a	No multivariate analysis performed so unclear if association of Mx/Mx_a with outcomes is mediated through ICP, CPP, admission GCS, age etc. Single institution dataset
Radolovich et al. (2011)	Primary: To assess the prognostic ability of Mx	Mx was strongly predictive of poor functional outcome (AUC = 0.69, *p* < 0.001)	Mx was predictive of poor functional outcome	No multivariate analysis performed so unclear if association of Mx with outcomes is mediated through ICP, CPP, admission GCS, age etc. Single institution dataset Significantly variable duration of insonation
Schmidt et al. (2016a)	Primary: To assess the association of Mx with functional outcomes following TBI Secondary: To assess the correlation of Mx with PRx	Mx correlated with 3-month GOS (r = −0.54, *p* < 0.005) Mx was higher in the non-survivor group compared to the survivor group (0.28 ± 0.4 vs. 0.03 ± 0.21, *p* = 0.04) PRx and Mx correlated (r = 0.56, *p* < 0.001)	Mx was associated with functional outcome and survival following TBI Mx and PRx correlate positively with one another	No multivariate analysis performed so unclear if association of Mx with outcomes is mediated through ICP, CPP, admission GCS, age etc. Single institution dataset Heterogenous patient population
Schmidt et al. (2016b)	Primary: Examine the association of Mx and PRx with mortality and functional outcomes Secondary: To identify a critical threshold for Mx	Mx was not significantly different between those that died in hospital and those that survived Mx and not PRx was significantly different between those with a good outcome vs. those with a poor outcome (−0.07 ± 0.21 vs. 0.21 ± 0.27, *p* < 0.005) In the subgroup of TBI patients PRx correlated with 3-month functional outcome (r = 0.63, *p* < 0.01) while Mx did not Mx of 0.2 was found to be a critical threshold for poor outcome (p <0.005)	Mx was not able to predict mortality and but was significantly different in those with good functional outcomes compared with those with poor functional outcomes Mx has a critical threshold of 0.2 for poor outcomes Mx was not associated with functional outcomes in the subgroup of TBI patients	No multivariate analysis performed Single institution dataset Heterogenous patient population
Sorrentino et al. (2011)	Primary: To identify a threshold value for Mx and Mx_a where survival and functional outcomes worsen Secondary: To assess the association of Mx and Mx_a with functional outcomes and each other	The threshold (by 2x2 chi square analysis) was 0.3 for survival and good functional outcome for Mx_a For Mx, 0.05 was found to be the threshold for good functional outcome while 0.3 was the threshold for survival Mx and Mx_a were both negatively correlated with outcomes (r = −0.2, *p* < 0.01 and r = −0.15, *p* = 0.01, respectively) Mx was significantly correlated with Mx_a (r = 0.78, *p* < 0.001)	Mx has a threshold for worsening survival at 0.3 while outcomes worsen above 0.05 Mx_a has a single threshold for worsening survival and poor outcomes at 0.3 Mx and Mx_a are correlated with one another and both are associated with functional outcome	No multivariate analysis performed so unclear if association of Mx with outcomes is mediated through ICP, CPP, admission GCS, age etc. Single institution dataset No evaluation of thresholds outside of dataset used to derive them
Zeiler et al. (2017b)	Primary: To assess the covariance of various indices of cerebrovascular reactivity Secondary: To assess the association of various indices of cerebrovascular reactivity and functional outcome	Mx correlated well with Dx (r= 0.991, *p* < 0.0001) and Sx (r = 0.726, p < 0.0001) Mx correlated moderately with PRx Sx and Sx_a correlated well with ICP based indices such as PRx Mx, Sx, and Dx were correlated with Mx_a, Dx_a and Sx_a strongly Dx and Dx_a only strongly correlated with Mx and Sx No statistically significant association with functional outcomes was found for any of the indices	CPP based TCD indices correlated well with one another Sx and Sx_a correlated most significantly with PRx CPP based and ABP based TCD indices correlate strongly No association with functional outcome was found likely due to small sample size	No multivariate analysis performed Single institution dataset Small patient population with limited recording time
Zeiler et al. (2018a)	Primary: To confirm the covariance of TCD and ICP derived indices such at PRx Secondary: To identify thresholds for Sx, Sx_a, Dx, and Dx_a associated with functional outcomes	Sx and Sx_a displays the strongest correlation with ICP based indices compared to other TCD based indices Principle Component Analysis also found Sx and Sx_a to be most associated with ICP based indices Sx and Sx_a also co-clustered with ICP based indices by Agglomerative Hierarchal Clustering and K-Means Cluster Analysis Sx was able to discriminate between alive/dead (AUC = 0.630, *p* = 0.005) and favourable/unfavourable (AUC = 0.646, *p* = 0.001) by univariate logistic regression and performed better than Dx and Mx Sx was found to have a critical threshold of −0.15 for unfavourable outcome (*p* < 0.001) and −0.20 for mortality (*p* < 0.0001) by sequential chi-square thresholding Sx_a was able to discriminate between alive/dead (AUC = 0.582, *p* = 0.068) and favourable/unfavourable (AUC = 0.632, *p* = 0.001) by univariate logistic regression and performed better than Dx_a and Mx_a Sx_a was found to have a critical threshold of −0.10 for unfavourable outcome (*p* = 0.0001) and +0.05 for mortality (*p* = 0.019) by sequential chi-square thresholding Chi-square values of Sx were higher than for Sx_a in thresholding analysis indicating a stronger relationship between thresholds and outcomes for Sx than Sx_a Dx was able to discriminate between favourable/unfavourable outcomes (AUC = 0.592, *p* = 0.012) but failed to reach significance when predicting mortality by univariate logistic regression Dx was found to have a threshold of −0.10 (*p* = 0.005) for unfavourable outcomes Dx_a failed to reach significance for 6-month outcomes and mortality	Sx and Sx_a seem to be the most strongly associated with ICP indices of all TCD based indices Sx outperformed Mx and Dx are discriminating between alive/dead and favourable/unfavourable outcomes Sx has a critical threshold at −0.15 for unfavourable functional outcome and −0.20 for mortality Sx_a outperformed Mx_a and Dx_a at discriminating between alive/dead and favourable/unfavourable functional outcomes Sx_a has a critical threshold at −0.10 for unfavourable outcome and +0.05 for mortality Sx was superior to Sx_a when predicting functional outcome Dx was only found to be able to discriminate between favourable and unfavourable outcomes with a threshold of −0.10 Dx_a failed to display any prognostic value	No multivariate analysis performed Single institution dataset
Cerebral physiologic correlates				
Cerebral physiologic correlatesCzosnyka et al. (2003)	Primary: To evaluate the relationship between Mx and ICP, CPP and ABP Secondary: The examine the association between hemispheric differences in Mx and imaging findings on admission and mortality	The relationship between Mx and ABP or CPP was U-shaped Mx was disturbed when CPP < 65 mmHg and > 90 mmHg and when ICP > 25 mmHg Mx was > 0.2 during plateau waves of ICP Mx was significantly worse on the side of the contusion and on the side of brain expansion in patients with MLS (*p* < 0.05) Hemispheric differences in Mx were more significant and more common in those that died in hospital	Mx can be used to identify a range of CPP or ABP over which cerebrovascular reactivity is most intact Cerebrovascular reactivity, as measured by Mx, is disrupted during plateau waves of ICP Hemispheric difference in injury can be detected by Mx and is prognostically relevant	No assessment of association of cerebrovascular reactivity and progression of radiographic findings Single institution dataset Heterogenous patient cohort including non-TBI patients
Haubrich et al. (2011)	Primary: To assess if CPPopt, as determined by Mx, was altered by hypocapnia Secondary: To assess if hypocapnia alters the ability to detect CPPopt with Mx	Hypocapnia improved cerebrovascular reactivity in those where it was impaired (Mx >= 0.25) (left: 0.33 ± 0.149; right: 0.37 ± 0.24 vs. left: 0.06 ± 0.27; right: −0.001 ± 0.17, *p* < 0.001) Hypocapnia did not change cerebrovascular reactivity in those where it was intact (Mx < 0.25) Hypocapnia increased the number of patients for which a CPPopt was identified from 12 to 29 There was no significant change in CPPopt at normocapnia or hypocapnia	In those with impaired cerebrovascular reactivity, hyperventilation improves Mx In those with intact cerebrovascular reactivity, hyperventilation did not significantly change Mx Hypocapnia seems to not affect CPPopt but may aid its detection	Single institution dataset Short recording period especially for determine a CPPopt
Haubrich et al. (2012)	Primary: To assess the impact of hypocapnia on Mx.	No significant change in Mx was seen with hypocapnia in those with intact cerebrovascular reactivity as defined by Mx < 0.25 In patients with impaired cerebrovascular reactivity, as defined by Mx > 0.25, hypocapnia significantly reduces Mx (0.37 ± 0.13 to 0.12 ± 0.25, *p* < 0.01)	In those with impaired cerebrovascular reactivity, hyperventilation improves Mx In those with intact cerebrovascular reactivity, hyperventilation did not significantly change Mx	Single institution dataset Short recording period
Lang (2003)	Primary: To assess the association of Mx with PRx.	There was a significant overall correlation between PRx and Mx (Pearson correlation, r = 0.42, *p* < 0.007, Spearman correlation, r = 0.43, *p* < 0.05)	Mx and PRx correlate with one another.	Single institution dataset No indication of duration of insonation
Lewis et al. (2014)	Primary: To evaluate the behavior of Mx during plateau waves of ICP	Mx during plateau wave was significantly more positive when compared to pre-plateau and post-plateau recordings (0.91 vs. 0.27 and 0.29, *p* = 0.001)	Cerebrovascular reactivity, as measured by Mx, is significantly disrupted during plateau waves of ICP.	Small number of events observed.
Liu et al. (2016)	Primary: To evaluate the behavior of Mx and Mx_a during plateau waves of ICP	Mx increased from 0.12 ± 0.40 at baseline to 0.47 ± 0.47 at plateau *p* = 0.004 No significant difference was found between Mx_a at baseline and at plateau	The deterioration of cerebrovascular reactivity during plateau waves of ICP was detected by Mx but not Mx_a	Small number of events observed
Schmidt et al. (2003a)	Primary: To assess the association of Mx with Mx_a	Mx and Mx_a correlated well with one another (r = 0.86, *p* < 0.001)	Mx and Mx_a correlate with one another	No indication of duration of insonation Heterogenous patient cohort including non-TBI patients
Schmidt et al. (2003b)	Primary: To assess the prognostic value of hemispheric asymmetry in Mx	Absolute left to right difference in Mx was correlated with Mx (mean left-right) (r = 0.24, *p* < 0.025) Asymmetry was higher in those that died than survived (0.14 ± 0.18 vs. 0.08 ± 0.1, *p* = 0.04) Mx was higher in those that died (0.13 ± 0.05 vs. −0.03 ± 0.05, *p* = 0.002) By multiple regression analysis outcome was independently correlated with asymmetry of Mx (*p* < 0.0015)	Hemispheric asymmetry in Mx was independently associated with outcome following TBI	Single institution dataset
Schmidt et al. (2009)	Primary: The assess if Mx/Mx_a were different during increases of CPP than during decreases of CPP	Mx was significantly different during increases of CPP than during decreases of CPP (0.05 ± 0.49 vs. 0.14 ± 0.54, *p* < 0.005) Mx_a was not significantly different during increases in CPP compared to decreases in CPP	Cerebrovascular reactivity, as measured by Mx, appears to be stronger during increases in CPP than during decreases in CPP	During analysis, a large proportion of data was discarded with data from 53 out of 210 patients used in the study
Schmidt et al. (2012)	Primary: To evaluate if cerebrovascular reactivity differs during increases in CPP as compared to decreases in CPP	Cerebrovascular reactivity, as measured by Mx, was stronger during increases in CPP compared to decreases in CPP (0.06 ± 0.52 vs. 0.15 ± 0.55, *p* < 0.005)	Cerebrovascular reactivity appears to be stronger during increases of CPP than during decreases of CPP	Data used in study represented only a small amount of original dataset due to limited availability of TCD recordings
Zeiler et al. (2018c)	Primary: To evaluate the association of various TCD based indices with ICP based indices, such as PRx	Mx and PRx were correlated (r = 0.346, *p* = 0.006) Sx and Sx_a were moderately correlated with ICP based indices such as PRx ICP based indices, such as PRx, were found to be most associated with Sx and Sx_a by Principal Component Analysis and by Agglomerative Hierarchal Clustering	PRx and Mx are correlated with one another Sx and Sx_a showed a stronger association with ICP based indices than other TCD based indices	Single institution dataset Short recording period
Zeiler et al. (2018e)	Primary: To determine if PRx can be estimated by Mx_a and Sx_a	The model of PRx using Sx_a correlated well with PRx (r = 0.794, 95% CI 0.788–0.799, *p* < 0.0001) The model of PRx using Sx_a and Mx_a correlated well with PRx (r = 0.809, 95% CI 0.809−0.819, *p* < 0.0001)	PRx can be estimated using ABP and TCD based indices Mx_a and Sx_a	Model development cohort was the same as the validation cohort Single institution dataset
Zhang et al. (2016)	Primary: To assess the association of Mx with CO2 reactivity Secondary: To assess the impact of Hyperventilation of Mx	CO2 reactivity was correlated with Mx (r = −0.37, *p* = 0.04) Mx did not significantly change with hyperventilation	Cerebrovascular reactivity, as measured by Mx, is associated with CO2 reactivity Cerebrovascular reactivity, as measured by Mx was not significantly altered by hyperventilation	Small sample size with limited recording time
Zweifel et al. (2008)	Primary: To assess the correlation between Mx and PRx	There was good correlation between Mx and PRx (r = 0.36, *p* < 0.001)	Both measures of cerebrovascular reactivity, Mx and PRx, correlated with one another.	No indication of duration of insonation TCD was not available for each patient Single institution dataset

ABP, arterial blood pressure; CBFV, cerebral blood flow velocity; CPP, cerebral perfusion pressure; CPPopt, optimal CPP, Dx, diastolic flow index with cpp, Dx_a, diastolic flow index with ABP; GCS, glasgow coma scale; GOS, glasgow outcome scale; ICP, intracranial pressure; Mx, mean flow index with cpp; MLS, midline shift; Mx_a, mean flow index with ABP, PRx, pressure reactivity index; Sx, systolic flow index with cpp; Sx_a, systolic flow index with ABP

### Mortality

There is strong evidence of an association between dysfunctional cerebrovascular reactivity, as measured by continuous time-domain TCD based indices, and mortality following TBI, with numerous studies finding a higher Mx correlating with mortality (Czosnyka et al., 2002; Schmidt. et al., 2003b; Schmidt. et al., 2016a; Budohoski et al., 2012a; Budohoski et al., 2012c). A notable exceptions to this was a 2016 study by Schmidt and colleagues which contained a mixed cohort of 20 TBI patients and 21 non-TBI patients (Schmidt et al., 2016b).

The evidence for the association between other TCD based indices and mortality is not as clear with a 2018 study by Zeiler and colleagues having most comprehensively examined this relationship in a cohort of 281 TBI patients (Zeiler et al., 2018a). Sx and Sx_a were found to have the strongest associations with mortality for indices with CPP and ABP as inputs, respectively, while Mx and Mx_a were also found to have some association. Neither Dx nor Dx_a were found to have any correlation with mortality.

### Global Functional Outcomes

Most studies chose to use Glasgow Outcome Scale (GOS) as their outcome metric of choice, while opting to dichotomize outcome into favourable/unfavourable or good/poor (with favourable typically denoted as GOS of 5 or above and unfavourable as GOS 4 or less). Follow up was usually collected at 6 months post-injury. Once again, Mx seems to have the strongest body of evidence supporting its association with outcomes with studies consistently finding that a higher Mx was associated with poor or unfavourable outcome at follow up (Czosnyka et al., 1996; Czosnyka et al., 1997; Czosnyka et al., 1999; Czosnyka et al., 2000; Czosnyka et al., 2001; Czosnyka et al., 2002; Czosnyka et al., 2008; Lang et al., 2003a; Lewis et al., 2007; Lewis et al., 2012; Radolovich et al., 2011; Sorrentino et al., 2011; Budohoski et al., 2012a; Budohoski et al., 2012c; Liu et al., 2015; Schmidt et al., 2016a). When Mx was found to not be associated with outcomes, it was often attributed to small sample sizes (Schmidt et al., 2016b; Zeiler et al., 2017b).

Once again, the evidence supporting other TCD based indices is not as prevalent. Mx_a was found to be associated with global functional outcomes in four different articles with higher values being associated with worse outcomes (Lang et al., 2003a; Sorrentino et al., 2011; Liu et al., 2015; Zeiler et al., 2018a). Dx and Dx_a have been more recently examined with Dx having a weak association with functional outcomes and Dx_a failing to demonstrate any predictive value. In the original 1996 study by Czosnyka and colleagues Sx was correlated with 6-month GOS (Czosnyka et al., 1996). More recently, Sx and Sx_a have been found to be the most strongly correlated with outcome of those indices with CPP and ABP as inputs, respectively, (Zeiler et al., 2018a). Of note, a 2005 study by Czosnyka and colleagues found that Sx was not associated with functional outcome when utilizing a multiple regression model including ICP and age (Czosnyka et al., 2005).

### Thresholds

Thresholds values of indices, where outcomes or mortality significantly increases, was the focus of 4 articles. In a 2002 study by Czonyka and colleagues they found a threshold for Mx of 0.23, above which mortality went from 11 to 47% (Czosnyka et al., 2002). A similar threshold for mortality, Mx = 0.3, was found in a follow up study by Sorrentino and colleagues in 2011. In that same study, a threshold of 0.05 for Mx was found to be where functional outcomes most drastically worsened while Schmidt and colleagues found a threshold for unfavourable outcomes closer to that of the mortality threshold at Mx = 0.2 (Sorrentino et al., 2011; Schmidt et al., 2016b).

In the 2011 study by Sorrentino and colleagues, Mx_a was found to have a threshold at 0.3 where both mortality and functional outcome both worsened in their cohort (Sorrentino et al., 2011). Zeiler and colleagues found in 2018 that in their cohort of 281 TBI patients, Sx had thresholds of −0.15 and −0.20 while Sx_a had thresholds of −0.10 and 0.05 for functional outcome and mortality, respectively. They also identified a threshold of −0.10 for Dx where outcomes worsened (Zeiler et al., 2018a).

### Hemispheric Asymmetry

TCD can be utilized to insonate the left and right MCA of the patient without any increased risk to the patient and so a number of studies examined the effect of hemispheric asymmetry of TCD based indices of cerebrovascular reactivity. Czosnyka and colleagues noted in their 2003 study that Mx was worse on the side of contusion or expansion if the patient had midline shift. They also noted that a hemispheric asymmetry was significantly more common in patients that died than those that survived (Czosnyka et al., 2003). In a follow up study Schmidt and colleagues found that the magnitude of hemispheric asymmetry, as determined by Mx, was higher in patients that died compared to those that survived and that hemispheric asymmetry was independently associated with functional outcome by multiple regression analysis (Schmidt et al., 2003b). Interestingly, hemispheric asymmetry, as determined by Mx_a was not found to be associated with outcomes by Lang and colleagues in their 2003 study (Lang et al., 2003b).

### Established Measure of Cerebrovascular Reactivity

Cerebrovascular reactivity is often measured by the fluctuation of ICP in response to changes in ABP in a more established index known as the pressure reactivity index (PRx). There has been a building body of evidence supporting cerebrovascular reactivity, in the form of PRx, being an important physiologic measure in TBI and so several studies have assessed the covariance of PRx and TCD based indices (Czosnyka et al., 1997; Czosnyka et al., 2002; Lang, 2003; Zweifel et al., 2008; Budohoski et al., 2012a; Schmidt et al., 2016a; Zeiler et al., 2017b; Zeiler et al., 2018a; Zeiler et al., 2018c). Once again, Mx has been the most well examined TCD based index with eight studies demonstrating some degree of correlation between Mx and PRx (Czosnyka et al., 1997; Czosnyka et al., 2002; Lang, 2003; Zweifel et al., 2008; Budohoski et al., 2012a; Schmidt et al., 2016a; Zeiler et al., 2017b; Zeiler et al., 2018c). Despite this, recent work comparing various TCD based indices found that Sx had the strongest correlation with PRx (Zeiler et al., 2017b; Zeiler et al., 2018a; Zeiler et al., 2018c), and both Sx and Sx_a appear to closely approximate PRx through advanced time-series modeling (Zeiler et al., 2018e). Finally, utilizing complex time-series analysis, PRx has been found to be estimated well with Mx_a and Sx_a based models (Zeiler et al., 2018e).

### Cerebral Perfusion Pressure and Intracranial Pressure

The relationship between TCD based indices and ICP seems to be somewhat independent with Mx being found to be positively correlated with ICP in early studies and a notable increased in Mx with ICPs above 20 mm Hg (Czosnyka et al., 1999; Czosnyka et al., 2001; Czosnyka et al., 2003; Czosnyka et al., 2008). Notably, during measurements of plateau waves of ICP, Mx was greater than the threshold of 0.2 and significantly more positive than pre-plateau and post-plateau periods (Czosnyka et al., 2003; Lewis et al., 2014). This difference during plateau waves was not found in Mx_a (Liu et al., 2016). The association between TCD indices and CPP is slightly more complex. While early studies showed a negative correlation between Mx and CPP(Czosnyka et al., 1999; Czosnyka et al., 2000) subsequent studies have found that when plotting Mx vs. CPP a U-shaped distribution is observed (Czosnyka et al., 2001; Czosnyka et al., 2003; Lewis et al., 2012). This may indicate that an optimal CPP (CPPopt) may exist where Mx is at a minimum, and therefore cerebrovascular reactivity may be best preserved, for individual patients. Cerebrovascular reactivity may then be disrupted both when CPP is inadequate or insufficient and this has been postulated as a means of identifying individual CPP targets based on a CPPopt derived from Mx. Interestingly, some studies have also found that Mx was significantly lower when CPP was increasing compared to when it was decreasing and that the correlation between Mx and CPP is stronger during increases in CPP than decreases in CPP (Schmidt et al., 2009; Schmidt et al., 2012). This may indicate that current management generally places patients at a CPP less than their CPPopt.

### CO_2_ Reactivity

A number of smaller studies have explored the relationship between TCD based indices and hypocapnia as well as CO_2_ reactivity (Haubrich et al., 2011; Haubrich et al., 2012; Zhang et al., 2016). In general, those with disrupted cerebrovascular reactivity (Mx ≥ 0.25), moderate hypocapnia seemed to significantly decreased Mx while those with intact reactivity (Mx ≤ 0.25) saw no significant change with hypocapnia (Haubrich et al., 2011, Haubrich et al., 2012). Of note, while less than half of the patients examined had an identifiable CPPopt at normocapnia, nearly all of them had one with hypocapnia (Haubrich et al., 2011). In a similar study, Zhang and colleagues found that CO_2_ reactivity was correlated with Mx but did not find any significant changes in Mx with hyperventilation (Zhang et al., 2016).

### Radiographic Evolution of Injury

No study was identified that examined the association between time-domain TCD based indices of cerebrovascular reactivity and the evolution of imaging findings following TBI.

## Discussion

A strong relationship between time-domain TCD based indices of cerebrovascular reactivity and mortality/functional outcome following TBI has been demonstrated through this scoping review of the literature (Czosnyka et al., 1996; Czosnyka et al., 1997; Czosnyka et al., 1999; Czosnyka et al., 2000; Czosnyka et al., 2001; Czosnyka et al., 2002; Lang et al., 2003a; Schmidt et al., 2003b; Lewis et al., 2007; Radolovich et al., 2011; Sorrentino et al., 2011; Budohoski et al., 2012a; Budohoski et al., 2012c; Lewis et al., 2012; Schmidt et al., 2016a). The prognostic utility of TCD based indices has been emphasized with the identification of thresholds for most indices at which outcomes worsen and mortality increases (Czosnyka et al., 2002; Sorrentino et al., 2011; Schmidt et al., 2016b; Zeiler et al., 2018a). Hemispheric asymmetry of cerebrovascular reactivity, as measured by TCD based indices, also seems to pertain a poor prognosis following TBI (Czosnyka et al., 2003; Lang et al., 2003b; Schmidt et al., 2003b). There also seems to be a reasonable degree of covariance between TCD based indices of cerebrovascular reactivity and more established measures, such as PRx (Czosnyka et al., 1997; Czosnyka et al., 2002; Lang, 2003; Zweifel et al., 2008; Budohoski et al., 2012a; Schmidt et al., 2016a; Zeiler et al., 2017b, Zeiler et al., 2018c). Elevations in ICP seem to be associated with the disruption of cerebrovascular reactivity (Czosnyka et al., 1999; Czosnyka et al., 2001; Czosnyka et al., 2003; Czosnyka et al., 2008) while a U-shape relationship exist between CPP and Mx indicating the possibility of a CPPopt where cerebrovascular reactivity is the least disrupted (Czosnyka et al., 2001; Czosnyka et al., 2003; Lewis et al., 2012). Finally, hyperventilation seems to improve cerebrovascular reactivity when it is already disrupted but does not significantly change it when already intact (Haubrich et al., 2011; Haubrich et al., 2012; Zhang et al., 2016). These findings highlight the utility of these indices in not only provided a greater understanding of the post-TBI physiome but also indicate a role for these measure of cerebrovascular reactivity in precision medicine by aiding in developing personalized targets for physiologic parameters following injury.

This review has also identified some major limitations to this body of literature. Perhaps, most obvious, is that the predominance of data supporting these findings comes from a single institution. This limits the confidence in the generalizability of these findings. A more subtle corollary of this is that there is significant overlap in cohorts used over various articles and so while there may be numerous publications finding prognostic utility in these indices, the strength of these finding may be somewhat overstated as the data is not wholly unique between them.

There are also limitations to the assessment of global outcomes measured at follow up. None of the studies identified in this scoping review utilized pathology specific, detailed quality of life measures such as the Quality of Life after Brain Injury (QOLIBRI) instrument. Assessments such as these would have provided a more comprehensive understanding of a patient’s health-related quality of life following their injury (von Steinbüchel et al., 2020). Additionally, trends in functional outcome, identified through serial measurements collected over multiple timepoints, were not investigated by any of the studies. As a result, no comments can be made about the utility of TCD based indices of cerebrovascular reactivity to stratify various trajectories of functional recovery.

Another weakness is in the analysis performed in these studies. We have seen that these indices are related to age, severity of injury and ICP (Czosnyka et al., 2000; Czosnyka et al., 2003; Czosnyka et al., 2005), however, what is unclear is if these indices provide prognostic utility independent of these associated variables as no multivariate analysis was performed. Without multivariate analysis, the role of TCD based indices as an independent prognostic tool remains unclear. Further to this, the studies identified reduced measures of TCD indices to grand averages over the recording period or over the length of stay with no study performing time-series analytics to evaluate causal relationships. While this simplifies analysis, it is at the cost information encoded in the fluctuations of these indices over a recording period or over the entire length of stay.

The literature also seems to mainly focus on Mx as the TCD based index most studied. This is likely due to it being the first TCD based index described (Czosnyka et al., 1996). While this means there is a large volume of evidence supporting its use, studies exploring other indices are limited. Of note, systolic TCD based indices (Sx and Sx_a) were only examined in seven studies (Czosnyka et al., 1996; Czosnyka et al., 2005; Budohoski et al., 2012c; Zeiler et al., 2017b; Zeiler et al., 2018a; Zeiler et al., 2018c; Zeiler et al., 2018e). This is especially unfortunate given recent studies finding systolic based indices to closest association with more established ICP based indices of cerebrovascular reactivity and provide better prognostic utility than mean flow (Mx and Mx_a) and diastolic (Dx and Dx_a) TCD based indices(Zeiler et al., 2018a). Diastolic TCD based indices (Dx and Dx_a) were only examined in four studies and were found to have the weakest outcome associations of all modalities (Budohoski et al., 2012c; Zeiler et al., 2017b; Zeiler et al., 2018a; Zeiler et al., 2018c). However, generalization of the findings of diastolic (Dx and Dx_a) and systolic (Sx and Sx_a) modalities are limited not only by the small number of studies but also due to the fact that all of the identified studies evaluating them draw from a single institution’s database (Addenbrooke’s Hospital, United Kingdom).

There are also some limitations inherent to TCD that become apparent when reviewing the literature. Due to the difficulty in obtaining prolonged recordings of TCD data, most studies report only data collection during a small proportion of time spent in ICU with some studies reporting as little as 10 min of insonation a day (Radolovich et al., 2011) and no study reporting more than 4 h per day. Given the dynamic nature of cerebrovascular reactivity, as demonstrated by the PRx literature (Adams et al., 2017), it is hard to believe that prognostic utility would not benefit from longer periods of insonation. In addition to the variable duration of insonation, there is also inconsistent timing and rate of measurements with some studies performing serial daily measurements (Czosnyka et al., 1996; Czosnyka et al., 1998; Czosnyka et al., 1999; Czosnyka et al., 2000; Czosnyka et al., 2001; Czosnyka et al., 2002; Czosnyka et al., 2003; Schmidt et al., 2003b; Czosnyka et al., 2005; Lewis et al., 2007; Czosnyka et al., 2008; Radolovich et al., 2011; Sorrentino et al., 2011; Schmidt et al., 2012; Liu et al., 2015) while others reported only performing one recording session over the course of admission (Lang et al., 2003b; Zeiler et al., 2018a; Zeiler et al., 2018c; Zeiler et al., 2018e). A number of studies also failed to report any details around duration and frequency of insonation as well as timing of measurement following injury(Czosnyka et al., 1997; Lang et al., 2003a; Lang, 2003; Schmidt et al., 2003a; Zweifel et al., 2008; Schmidt et al., 2009; Budohoski et al., 2012a; Budohoski et al., 2012c; Lewis et al., 2012; Lewis et al., 2014; Schmidt et al., 2016a; Schmidt et al., 2016b; Liu et al., 2016; Zhang et al., 2016). In those studies, in which serial assessments were performed, no comment was made on the progression of cerebrovascular reactivity over time.

Finally, there seems to be an absence of any literature evaluating the link between cerebrovascular reactivity, as measured by time-domain TCD based indices, and the evolution or progression of imaging findings in the setting of TBI. Similar reports have been published with regards to PRx and have found a link between cerebrovascular reactivity and lesion progression following TBI (Mathieu et al., 2020a; Mathieu et al., 2020b; Zeiler et al., 2020). A link between similar TCD based indices may provide a less invasive means of predicting radiographic progression and should be explored further.

### Limitations

This scoping review does, in and of itself, has some limitations. Articles included in this study were limited to those that contained cohorts of greater than 20 TBI patients. This was done in order to limit the prevalence of small case series and case reports, however, this may have limited the diversity of institutions included in this review. Additionally, this review was limited to continuous time-domain TCD based indices at the exclusion of frequency domain based TCD indices. While this was done to avoid excessive heterogeneity in indices examined, there is evidence that these indices do associate with outcomes following TBI, but to a lesser degree (Liu et al., 2015). Additionally, the inclusion articles in journal supplements resulted in some very similar reports being included in this review (Haubrich et al., 2011; Haubrich et al., 2012). Finally, given that TBI has a global burden of disease, the exclusion of non-English language articles may have further narrowed the institutional diversity as well as likely skewed the ethnocultural diversity of the patient cohorts included in this review.

### Future Directions

This review highlights some key areas for further development. First, before TCD based indices are adopted clinically, validation of these findings will need to occur on more diverse datasets. Large multi-institutional/multi-national collaborative networks such as the ones found in the CENTER-TBI study and CAnadian High-Resolution TBI (CAHR-TBI) Research Collaborative are ideal for such validation studies (Bernard et al., 2020). In order to identify the true prognostic utility of TCD based indices, these studies should ideally examine the unique contributions of these indices in and above ICP, age and injury severity through multivariant analysis.

The clinically applicability of these indices may also be expanded past prognostication. Further exploration of utilizing TCD based indices to identify personalized CPP targets in critically ill patients following TBI must be undertaken. Precision medicine with personalized targets derived from these indices may provide the means to significantly alter outcomes following TBI and is already being explored utilizing more established measure of cerebrovascular reactivity, such as PRx (Zeiler et al., 2019a).

Further investigation of TCD based indices, aside from Mx, should also be done given the evidence of the strength of correlation with outcome Sx has demonstrated. Additionally, ABP based indices are of particular interest given the recent description of entirely non-invasive methods of collecting the requisite physiologic data (Zeiler et al., 2018b; Zeiler et al., 2019b; Zeiler and Smielewski, 2018; Gomez et al., 2020).

Finally, advances in TCD technology, such as robotic TCD has allowed for prolonged recording in and above 4 h due to the continuous robotic optimization of probe position. This has already been described as a means of assessing cerebrovascular reactivity for extended periods of time and may extend the percentage of time monitored while in ICU (Zeiler et al., 2018b; Zeiler and Smielewski, 2018). This may prove to further improve the association of these indices with outcomes following TBI while providing a more complete picture of the post-TBI physiome.

## Conclusion

This systematic scoping review of the literature identifies that there is a substantial body of evidence that cerebrovascular reactivity as measured by time-domain TCD based indices have prognostic utility following TBI. To a lesser extent, the literature has also explored some associations between these indices and cerebral physiologic parameters in this patient population. Notably, there is lack of evidence evaluating the correlation between these indices and radiographic progression following injury. Further research needs to be done to expand the generalizability of these results and identify optimal inputs and collection methods for these indices before they can be widely clinically adopted. However, their role in precision medicine following traumatic brain injury is promising.
