# Supplementary material for: Transcranial Doppler Based Cerebrovascular Reactivity Indices in Adult Traumatic Brain Injury: A Scoping Review of Associations With Patient Oriented Outcomes
Source: Front Pharmacol. 2021 Jul 6;12:690921. doi: 10.3389/fphar.2021.690921 (PMC8290494; doi:10.3389/fphar.2021.690921)
Supplement: Supplementary file 1 [file DataSheet1.docx]

**Supplementary Material**

**Appendix A: Search Strategy for SCOPUS Database**

**(KEY(“Traumatic Brain Injuries”) OR KEY(“Traumatic Brain Injury”) OR KEY(“TBI”) OR KEY(“Brain Trauma”) OR KEY(“Brain Traumas”) OR KEY(“Traumas, Brain”) OR KEY(“Trauma, Brain”) OR KEY(“Brain Injury, Traumatic”) OR KEY(“Encephalopathy, Traumatic”) OR KEY(“Encephalopathies, Traumatic”) OR KEY(“Traumatic Encephalopathies”) OR KEY(“Traumatic Encephalopathy”) OR KEY(“Injury, Brain, Traumatic”) OR KEY(“Closed head injury”) OR KEY(“Head injury”) OR KEY(“Head trauma”) OR KEY(“Traumatic subarachnoid hemorrhage”) OR KEY(“Traumatic subdural hematoma”) OR KEY(“Acute Subdural hematoma”) OR KEY(“Subdural Hematomas, Acute”) OR KEY(“Hematoma, Acute Subdural”) OR KEY(“Traumatic epidural hematoma”) OR KEY(“Cranial epidural hematoma”) OR KEY(“Epidural hematoma”) OR KEY(“Acute Epidural hematoma”) OR KEY(“Cranial Extradural Hematoma”) OR KEY(“Traumatic intracranial hemorrhage”) OR KEY(“Cranial Extradural Hematoma”) OR KEY(“Traumatic brain hemorrhage”) OR KEY(“Traumatic cerebral hemorrhage”) OR KEY(“Craniocerebral trauma”) OR KEY(“Decompressive craniectomy”) OR KEY(“Cerebral contusion”) OR KEY(“Frontal contusion”) OR KEY(“Temporal contusion”) OR KEY(“Brain contusion”) OR KEY(“Diffuse axonal injury”))**

**AND**

**(KEY(“Transcranial Doppler”) OR KEY(“TCD”) OR KEY(“Transcranial Doppler Sonography”) OR KEY(“Sonography, Doppler Transcranial”) OR KEY(“Ultrasonography, Doppler, Transcranial”) OR KEY(“Transcranial doppler ultrasound”) OR KEY(“Transcranial doppler ultrasonography”) OR KEY(“Cerebrovascular sonography”) OR KEY(“Cerebrovascular ultrasound”) OR KEY(“Cerebral blood Flow velocity”) OR KEY(“Neurosonology”) OR KEY(“Neurosonography”) OR KEY(“Neurosonologies”))**

**AND**

**(KEY(“Mx”) OR KEY(“Mxa”) OR KEY(“Mx_a”) OR KEY(“Sx”) OR KEY(“Sxa”) OR KEY(“Sx_a”) OR KEY(“Dx”) OR KEY(“Dxa”) OR KEY(“Dx_a”) OR KEY(“Cerebrovascular reactivity”) OR KEY(“Cerebral autoregulation”) OR KEY(“Autoregulation”) OR KEY(“Vascular reactivity”))**
